# Mouse Tracking to Explore Motor Inhibition Processes in Go/No-Go and Stop Signal Tasks

**DOI:** 10.3390/brainsci10070464

**Published:** 2020-07-20

**Authors:** Viola Benedetti, Gioele Gavazzi, Fabio Giovannelli, Riccardo Bravi, Fiorenza Giganti, Diego Minciacchi, Mario Mascalchi, Massimo Cincotta, Maria Pia Viggiano

**Affiliations:** 1 Section of Psychology—Department of Neuroscience, Psychology, Drug Research and Child’s Health (NEUROFARBA), University of Florence, 50135 Florence, Italy; viola.benedetti@stud.unifi.it (V.B.); fabio.giovannelli@unifi.it (F.G.); fiorenza.giganti@unifi.it (F.G.); 2 IRCCS SDN, 80142 Naples, Italy; gioelegavazzi@gmail.com; 3 Department of Experimental and Clinical Medicine, University of Florence, 50134 Florence, Italy; riccardo.bravi@unifi.it (R.B.); diego.minciacchi@unifi.it (D.M.); 4 Department of Experimental and Clinical Biomedical Sciences “Mario Serio”, University of Florence, 50134 Florence, Italy; mario.mascalchi@unifi.it; 5 Unit of Neurology of Florence, Central Tuscany Local Health Authority, 50143 Florence, Italy; massimo.cincotta@uslcentro.toscana.it

**Keywords:** proactive inhibition, reactive inhibition, go-no-go, stop signal task, mouse tracking, motor control, velocity profile

## Abstract

Response inhibition relies on both proactive and reactive mechanisms that exert a synergic control on goal-directed actions. It is typically evaluated by the go/no-go (GNG) and the stop signal task (SST) with response recording based on the key-press method. However, the analysis of discrete variables (i.e., present or absent responses) registered by key-press could be insufficient to capture dynamic aspects of inhibitory control. Trying to overcome this limitation, in the present study we used a mouse tracking procedure to characterize movement profiles related to proactive and reactive inhibition. A total of fifty-three participants performed a cued GNG and an SST. The cued GNG mainly involves proactive control whereas the reactive component is mainly engaged in the SST. We evaluated the velocity profile from mouse trajectories both for responses obtained in the Go conditions and for inhibitory failures. Movements were classified as one-shot when no corrections were observed. Multi-peaked velocity profiles were classified as non-one-shot. A higher proportion of one-shot movements was found in the SST compared to the cued GNG when subjects failed to inhibit responses. This result suggests that proactive control may be responsible for unsmooth profiles in inhibition failures, supporting a differentiation between these tasks.

## 1. Introduction

Motor inhibition reflects the ability to withhold a ‘prepotent’ response tendency and suppress inappropriate actions; it represents a core aspect of cognitive control allowing a flexible and efficient regulation of goal-directed behavior in daily life [1]. Motor inhibition relies on both proactive and reactive mechanisms that exert a synergic control on behavior [2]. Proactive inhibition refers to the ability to stand ready to inhibit an action in order to prevent inadequate behaviors, whereas reactive inhibition allows cancellation of a planned action in response to an external signal [3]. The functional neuroimaging literature supports the view that brain networks involved in proactive and reactive inhibition are distinct but partially overlapped [4].

Behavioral evaluation of response inhibition is typically performed using the go/no-go (GNG) or the stop signal task (SST) experimental paradigms. Although often used interchangeably, the GNG and the SST might investigate different components of the motor inhibitory function [5,6]. In fact, the GNG mainly involves proactive mechanisms engaged during the motor preparation, namely before the appearance of a target stimulus, reflecting the active maintenance of task goals [7]. In contrast, the SST mainly engages the reactive component, as the cancellation of an already initiated motor response is required after the stop signal appearance [8,9,10,11].

Behavioral performance is typically quantified by a key-press method [12,13]. However, this approach could be inadequate to capture sub-threshold responses [14,15,16,17,18]. For example, in a recent fMRI study on the relationship between impulsivity traits and proactive motor control, an activation of the primary motor cortex was observed during a GNG task when subjects correctly withheld responses (i.e., did not press the button) on No-Go trials [19]. This activation has been interpreted as due to sub-threshold responses (i.e., errors corrected ‘in flight’ or partial errors). Consistently, other studies conducted with different methods—including EMG and/or dynamometer [15,20,21]—have shown sub-threshold responses in similar and different cognitive domains.

An experimental approach which contributed to the shedding of light on the dynamic nature of cognitive processes is based on the analysis of continuous trajectories of hand movements in behavioral tasks [22,23]. In this framework, mouse-tracking procedures have been used to investigate different domains such as language, decision-making, learning and social cognition (see Freeman [24] for a review). Moreover, recent studies evaluated different mouse movement measures (e.g., velocity or acceleration in the mouse-cursor motion) obtained during GNG and SST tasks in patients with attention-deficit/hyperactivity disorder [25,26]. In this perspective, the characterization of movement trajectories by mouse-tracking procedures appears as a potentially valuable tool to explore the respective contribution of proactive and reactive mechanisms in response inhibition [24,27].

In the current study, we used a behavioral method based on a mouse response-registration system to investigate proactive and reactive components of motor inhibition during cued GNG and SST tasks. Velocity profiles extrapolated from mouse trajectories were recorded and the proportion of motor responses without trajectory corrections (defined as ‘one-shot-movements’ [28,29,30,31]) was evaluated either when subjects correctly responded in the Go conditions or when they failed to inhibit responses in No-Go/Stop conditions of both tasks. Motor control theories suggest that a point-to-point planned movement has no trajectory corrections unless specific control processes occurring during movement preparation are required [32,33]. Accordingly, we hypothesize that different movement profiles could be associated with inhibitory failures in GNG and SST paradigms, reflecting the influence of proactive and reactive mechanisms on motor preparation and execution.

## 2. Materials and Methods

### 2.1. Participants

A total of fifty-three healthy volunteers (37 women; mean age 24 years; range 18–40) with no history of neurological and psychiatric diseases or drug abuse, normal hearing and normal or corrected-to-normal vision were included in the study. All participants, but five, were right-handed. No participant was currently or previously engaged in sport activity at a competitive level or professional gaming. Participants were mainly recruited from the Psychology students’ community of the University of Florence. All participants gave their written informed consent to the procedure and the processing of personal data. The study was performed according to the Declaration of Helsinki and was approved by the Ethical Committee of the University of Florence (protocol number 63; date of approval: 23 January 2020). Prior to the experimental session, each subject was blind to the purpose of the study, which was carefully explained after the completion of the evaluation.

### 2.2. Experimental Procedure

Each participant performed the cued GNG [34] and the SST [9,35] in order to evaluate proactive and reactive processes, respectively. Subjects were positioned 57 cm away from the computer screen. OpenSesame 3.2.6 Kafkaesque Koffka [36] was used for stimuli production and response recording. The experiment took place in a quiet room, with poor lighting (no artificial light and reduced external light in order to obtain a semi-darkened room).

The order in which the tasks were performed by subjects was randomized and counterbalanced across subjects. Each task was immediately preceded by a training session to familiarize subjects with experimental procedures. It consisted of five sample trials for each task.

**Figure 1:**
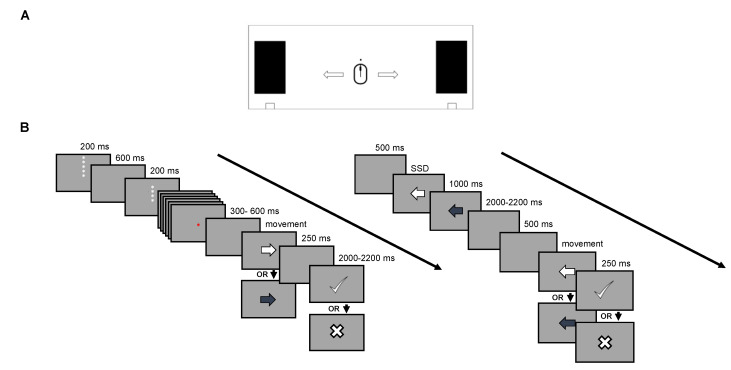
Mouse tracking system and experimental paradigms. (**A**) The system consists of a mouse device positioned in the board center. To emit a response, subjects were instructed to move the mouse in parallel to the x-axes of the board as quickly and accurately as possible in the direction indicated from the Go-stimulus (i.e., left or right), until they reached the barrier, bumping against it. After the response, the mouse had to be put back in the center. To inhibit a response, subjects were instructed to not move the mouse from the center of the board; (**B**) Trial structure of the cued go/no-go (GNG) is presented on the left. At the beginning of each trial, a descending series of five asterisks was presented, the latter three asterisks provided information about the probability that a ‘Go-stimulus’ or ‘No-Go-stimulus’ was presented (green asterisks = high ‘Go-stimulus’ probability, red asterisks = low ‘Go-stimulus’ probability). Subsequently, the target stimulus appeared, and subjects had to emit or inhibit (white arrow = respond, blue arrow = inhibit) the response. After correct responses, feedback about response speed was given (check mark = sufficient speed, X mark = too slow). Trial structure of the stop signal task (SST) is presented on the right. A white arrow was always presented at the beginning of each trial, and subjects were instructed to emit a response. Only on a minority of trials, after a variable amount of time, a blue arrow appeared and participants were instructed to inhibit the action, overriding the previous instruction. For correct responses, feedback about response speed was given, as for the cued GNG.

For both tasks, motor responses were collected using an optical gaming mouse-peripheral (KEY IDEA, model G10S, dimensions 4.84 × 2.64 × 1.53; weight 136 g). The mouse was positioned on the center of a wooden board delimited by two 28 × 10 cm sponges (Figure 1A). Each sponge was positioned at 12.5 cm from the center of the wooden table. The wooden board measured 30 × 70 cm and was anchored to the table by metal clamps to ensure stability (Figure 1A).

A preferential daily use of the right hand for a mouse device was reported also by the five left-handed subjects. Therefore, in both tasks all participants were instructed to respond with their right hand, regardless of their handedness.

#### 2.2.1. Proactive Inhibition (Cued GNG Task)

Visual stimuli consisted of arrows presented at the center of the screen (4 × 4 cm, ~4° of visual angle) (Figure 1B). Subjects were instructed to move the mouse as quickly and accurately as possible in the direction indicated by a ‘go’ target (white arrow) until they reached the sponge barrier, and to suppress the response when a ‘no-go’ target (blue arrow) was presented. Both ‘Go-stimulus’ and ‘No-Go-stimulus’ disappeared when the sponge barrier was reached or once 1000 ms was passed.

A descending series of five asterisks was presented at the beginning of each trial as a countdown to prepare the participant for the proper stimulus. This procedure was employed in order to heighten the proactive preparatory phase [19]. For ‘Go-stimulus’ trials the first asterisk was presented immediately after the feedback elapsed; for ‘No-Go-stimulus’ trials it was presented immediately after the maximum response time elapsed for correctly inhibited responses and after the threshold was reached for erroneous responses [19]. Each asterisk remained on screen for 200 ms and, between an asterisk and the following, a blank was presented for 600 ms. The countdown took 3400 ms to be fully displayed. The color of the last three asterisks during the countdown provided information on the probability that a ‘Go-stimulus’ or ‘No-Go-stimulus’ were presented. Namely, in the ‘high Go-stimulus probability’ condition (green asterisks) Go-stimuli were 70% likely (56 trials), whereas in the ‘low Go-stimulus probability’ condition (red asterisks) Go-stimuli were 30% likely (24 trials). Subjects were informed about the association between asterisk color and relative Go or No-Go stimulus probability.

In order to evaluate the role of cueing during the preparatory phase on subsequent movement profiles, we used the cued version of the GNG task, manipulating the probability that the upcoming target required a response (i.e., high vs. low Go stimulus probability).

The time between the end of the countdown and the appearance of the target varied randomly between 300 and 600 ms.

The order of ‘Go-stimulus’, ‘No-Go-stimulus’ and relative asterisk countdown trials was randomized for each participant. The task consisted of 160 trials.

See Video S1 (Supplementary Material) for an example of the task.

#### 2.2.2. Reactive Inhibition (Stop Signal Task)

The SST paradigm included two conditions: ‘Go-trials’ and ‘Stop-trials’ (Figure 1B). Each trial started with a fixation point presented at the center of the screen for 500 ms. Visual stimuli consisted of arrows presented at the center of the screen (4 × 4 cm, ~4° of visual angle). In Go-trials, a white arrow pointed randomly toward left or right. Subjects were instructed to move the mouse in parallel to the x-axes of the board as quickly and accurately as possible in the direction indicated by the arrow until they reached the sponge barrier. These trials represented 70% of the total trials (56 left-arrow and 56 right-arrow trials).

In Stop-trials (30% of the total trials), the white arrow was followed by a blue arrow (stop-signal) pointed in the same direction. Subjects were instructed to refrain from responding or to suppress the on-going motor response when the stop-signal was presented. The blue arrow disappeared after 1000 ms or as soon as the subject failed to inhibit a motor response (i.e., responses in which the mouse reached the sponge barrier). The time between the white and the blue arrows (Stop Signal Delay, SSD) was adapted to the participant’s performance by a tracking procedure [6,37,38,39,40]: when the subject succeeded correctly in inhibiting the response in Stop-trials, the SSD increased by 50 ms; when the subject failed to inhibit the SSD was shortened by 50 ms. The starting SSD value was individually set based on a 20-trial simple choice reaction time (CRT) test performed by each participant before the SST. Therefore, in the first trial of the SST, the SSD was the mean response time obtained at the CRT test minus 200 ms [41]. This value was set at the beginning of the experimental phase.

The inter-trial interval was varied randomly between 2000 and 2200 ms. The order of the Go-trials and Stop-trials was randomized for each participant. The task consisted of a total of 160 trials divided into two blocks.

See Video S2 (Supplementary Material) for an example of the task.

#### 2.2.3. Feedback on Response Speed

In both tasks, feedback on the response speed was given after ‘Go-conditions’ (i.e., Go-stimulus of the cued GNG and Go-trials of the SST), in order to limit the slowing tendency which can be adopted by the participant as a strategy to improve accuracy [41,42]. Namely, negative feedback (white X mark) was displayed for response trials with slow reaction times and participants were instructed to speed up the response in the subsequent trial. When subjects reached the sponge barrier within a pre-set time, a check mark (positive feedback) appeared on the screen. The feedback remained on the screen for 250 ms. The maximum response time after which the negative feedback was provided was the mean response time obtained at a simple CRT test minus one standard deviation. This procedure allowed the use of a stringent but realistic time response threshold reflecting individual differences in the speed of processing and response [41]. The feedback was provided only on response speed for ‘Go-conditions’, subjects did not receive feedback on their performance accuracy in terms of correct or erroneous responses.

### 2.3. Data Analysis

For each task and subject the behavioral performance was firstly quantified by the following measures: number of correct responses and reaction times (RT) in the Go conditions (Go-stimulus and Go-trials for GNG and SST, respectively), number of inhibitory failures in the No-Go/Stop conditions (i.e., No-Go-stimulus and Stop-trials, respectively), and the SSD (only for the SST). For the cued GNG, correct responses and inhibitory failures were calculated as total and as a function of the Go-stimulus probability (low and high probability). RTs were measured as the time between the stimulus appearance and the mouse movement onset. Movement onset was defined as the moment in which the subject exceeded a pre-determined threshold value (i.e., 30 pixels from the starting position). A mouse shift within 30 pixels was considered as device error. These values were chosen on the basis of preliminary recordings performed to calibrate the experimental apparatus. More precisely, one experimenter (V.B.) had to hold the mouse (230 Dots Per Inch —sampling rate 500 Hz) trying to stay as still as possible for 5 min. The farthest value obtained on the x-axes by the mouse was registered. After this, another session was performed. In an interval of time of 5 min the same experimenter had to keep the mouse still, alternating this condition with some random mouse movements. The maximum x values reached were measured. The maximum value measured between the two sessions was 30 pixels (3 mm) and it was chosen as the threshold to prevent ‘false positive’ movements. For both tasks, all mouse movements exceeding this threshold value of 30 pixels from the starting position were considered as inhibitory failures.

To evaluate the overall behavioral performance, the percentage of correct responses in the Go conditions were entered in a repeated-measures analysis of variance (ANOVA) with TASK as a within subject factor (three levels: cued GNG ‘high GO-stimulus probability’, cued GNG ‘low GO-stimulus probability’, and SST). Moreover, errors rates (inhibitory failures) during high and low GO-stimulus probability of the cued GNG were compared by a paired sample *t*-test.

**Figure 2:**
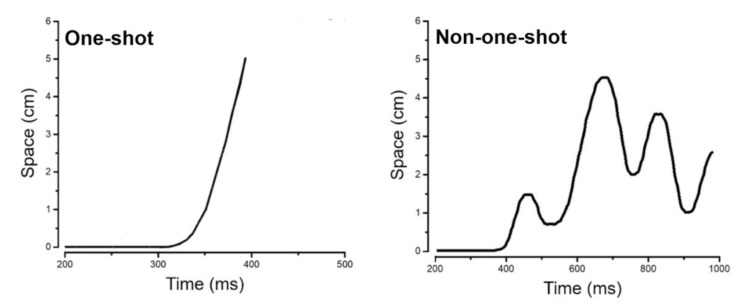
Movements profiles from one representative subject. A sample one-shot movement profile is presented on the displacement-time graph on the left. It consists of a steep slope without any peaks that reflects a smooth movement without motor command alteration. A sample non-one-shot movement profile is presented on the displacement-time graph on the right. It consists of a multi-peaked velocity profile reflecting motor command alteration.

As the main variable of interest, we evaluated the velocity profile extrapolated from the mouse trajectories both for correct responses obtained in the Go conditions and inhibitory failures of the No-Go/Stop conditions. We classified movements as one-shot or non-one-shot by visually inspecting each velocity profile. More precisely, a MATLAB code was used to automate the velocity profile computation and plotting to visually inspect the presence of trajectory corrections [29,30]. Movements were classified as one-shot when no correction was observed in the velocity profile (Figure 2). In contrast, trajectories with one or multiple corrections were considered as non-one-shot movements (Figure 2).

The proportions of one-shot movements calculated for either Go and No-Go/Stop conditions of both tasks were entered in separate repeated-measures ANOVA with TASK as a within subject factor (three levels: cued GNG ‘high GO-stimulus probability’, cued GNG ‘low GO-stimulus probability’, and SST).

Moreover, RTs recorded for Go and No-Go/Stop (inhibitory failures) conditions of both tasks were separately analyzed as a dependent variable using a mixed-model ANOVA with TASK (three levels: cued GNG ‘high GO-stimulus probability’, cued GNG ‘low GO-stimulus probability’, and SST) and VELOCITY PROFILE (two levels: one-shot and non-one-shot movements) as fixed factors. In the analyses conducted on RTs, participants were treated as a random factor. A mixed-models approach was chosen for its flexibility to efficiently handle designs that are not perfectly balanced (as in this case), taking into account the intrinsic (and uncontrolled) variability among the participants [29,30].

Finally, to evaluate whether the variation in individual SSD in the SST could have influenced the velocity profile, we conducted correlational analyses. Namely, the relationship between individual SSD values and the proportion of one-shot movements was tested by calculating the Pearson’s correlation coefficients separately for inhibitory failures and correct responses. SSD values were evaluated as follow: mean SSD; final SSD; and the difference between initial SSD and final SSD.

All tests were two-tailed and significance was set at *p* < 0.05 and adjusted by Bonferroni-correction for multiple comparisons. Partial eta squared (η_p_^2^) was calculated as effect size. In addition, a posteriori power analyses were performed using G*Power 3.1 software.

## 3. Results

### 3.1. Behavioural Performance

Details on behavioral performance in the cued GNG and in the SST are given in Table 1. As expected, task performance in the Go conditions was accurate as revealed by the percentage of correct responses with no significant differences between the tasks: F(2, 104) = 0.366, *p* = 0.695, η_p_^2^ = 0.007). The proportion of inhibitory failures during for the ‘high GO-stimulus probability’ was significantly higher compared to the ‘low GO-stimulus probability’ condition (t(52) = 2.371, *p* = 0.02).

### 3.2. Movement Profiles

The mean of one-shot movements calculated for either Go or No-Go/Stop conditions for both tasks are given in Table 2.

Results of the repeated measures ANOVA performed for the mean of one-shot movements during Go conditions did not show significant differences (F2, 104 = 2.321, *p* = 0.103, η _p_^2^ = 0.043, power = 0.17).

**Figure 3:**
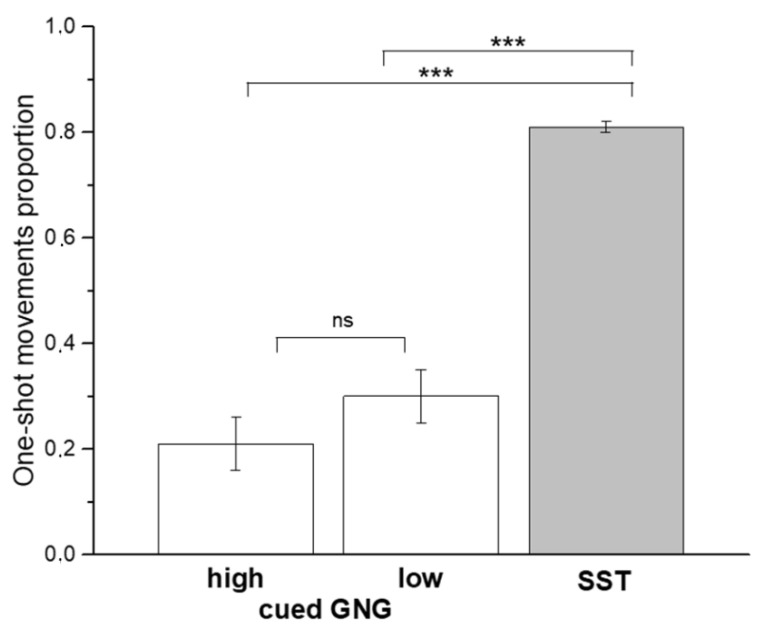
Proportion of one-shot movements. Proportion of one-shot movements evaluated during No-go/Stop conditions (inhibition failures) in the two tasks, averaged across subjects. Error bars represent ± 1 standard error of the mean. Asterisks mark a significant difference, *** *p* < 0.001 (Bonferroni-corrected).

In contrast, the mean of one-shot movements evaluated during No-go/Stop conditions (inhibition failures) differed significantly among tasks (F2, 104 = 67.981, *p* < 0.001, η _p_^2^ = 0.567, power = 0.99). Post-hoc comparisons (Bonferroni corrected) revealed that the mean of one-shot movements for either ‘high GO-stimulus probability’ or ‘low GO-stimulus probability’ conditions were significantly lower compared to those observed in the SST (*p* < 0.001) (Figure 3). In contrast, no significant differences emerged between ‘high GO-stimulus probability’ and ‘low GO-stimulus probability’ conditions (*p* = 0.652).

### 3.3. Reaction Times

RTs for one-shot and non-one-shot movements in either Go or No-go/Stop conditions (inhibition failures) are reported in Table 3.

For RTs recorded during Go conditions, the mixed-model ANOVA showed a significant main effect of TASK (F2,52 = 3.112, *p* = 0.048, power = 0.99), whereas the main effect of VELOCITY PROFILE (F1,52 = 0.631, *p* = 0.429, power = 0.51) and the interaction between these two fixed factors were not significant (F2,52 = 0.258, *p* = 0.772, power = 0.06).

**Figure 4:**
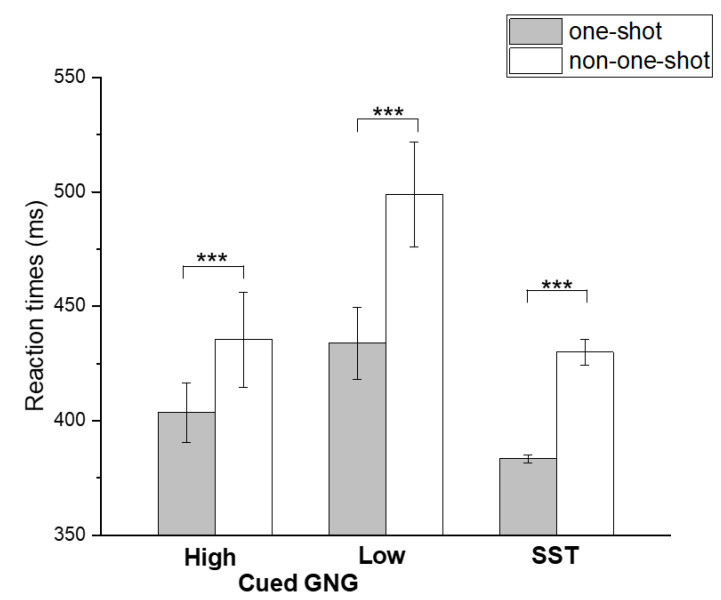
Reaction times. Mean reaction times (RTs) evaluated during No-go/Stop conditions (inhibition failures) in the two tasks, averaged across subjects. Error bars represent ± 1 standard error of the mean. Asterisks mark a significant difference, *** *p* < 0.001 (Bonferroni-corrected).

The mixed-model ANOVA conducted on RTs recorded during No-Go/Stop conditions (inhibitory failures) revealed a significant main effect of either TASK (F2,52 = 13.507, *p* < 0.001, power = 0.99) or VELOCITY PROFILE (F1,52 = 48.430, *p* < 0.001, power = 0.99), whereas the interaction between these two fixed factors was not significant (F2,52 = 0.080, *p* = 0.923, power = 0.05). Overall, when subjects failed to inhibit responses (inhibitory failures), RTs recorded during non-one-shot movements were significantly slower compared to those recorded during one-shot movements (Figure 4). Post-hoc comparisons (Bonferroni corrected) revealed that in Go conditions, the RT measured for ‘high GO-stimulus probability’ was faster than in ‘low GO-stimulus probability’ and SST conditions (*p* < 0.001). Concerning No-Go/Stop conditions, post-hoc comparisons revealed that the RT measured in SST one-shot movements was significantly faster than in all the other conditions, except for the RT measured in one-shot movements of ‘high GO-stimulus probability’ (all *p* < 0.001). Additionally, the RT measured in non-one-shot movements was slower than in all the other conditions (*p* < 0.001).

With regard to the relationship between individual SSD values in the SST and the proportion of one-shot movements, none of the correlations performed resulted statistically significant for either inhibitory failures or for correct responses (*p* values ranging between 0.112 and 0.811).

## 4. Discussion

In the current study we employed a mouse-tracking procedure to characterize the movement profile of fast hand motor responses recorded during the cued GNG and SST. We found a significantly higher proportion of one-shot movements in the SST compared to either conditions of the cued GNG when subjects failed to inhibit responses, whereas no differences emerged for movements executed in the Go conditions. Moreover, we found significantly slower RTs for non-one-shot movements compared with one-shot movements, independent of the task.

It is well-known that unconstrained point-to-point rapid hand movements are characterized by roughly straight trajectories, with typical bell-shaped velocity profiles, regardless of the amplitude and direction of the movement [32,33,43,44,45,46,47,48]. Neural processes during movement preparation influence different aspects of movement execution [49,50,51]. Therefore, distinctive smooth trajectories observed in rapid movements classified as one-shot may suggest that the influence of inhibitory control processes on motor planning may be absent or marginal. In particular, if a movement fails to be withheld and anything occurs between its planning and its execution, it is conceivable that neither proactive nor other cognitive processes are successfully intervening over the motor control processes.

Our analysis of velocity profiles revealed that inhibitory failures in the cued GNG task were more frequently associated with adjustments of the initial motor plan with respect to the trajectories performed during the SST. This difference may be interpreted according to the prevalent inhibitory component engaged by the task employed. In fact, when the inhibitory mechanisms engaged were mainly reactive (as in the SST), trajectory corrections to the initial motor plan observed for inhibitory failures were less frequent. In contrast, the opposite trend emerged when the inhibitory demand was mainly proactive (as in the cued GNG). Moreover, the probability that the upcoming target required a response did not affect the subsequent movement profiles as no significant differences in the proportion of one-shot movements emerged between the high and low Go stimulus probability conditions of the cued GNG task, neither in the Go nor in the No-Go conditions. However, the possibility that the cued procedure employed (not using a block-wise design) may actually have reduced the error, effectively reducing the distinguishable difference between the two probability conditions, cannot be ruled out.

We speculate that the higher proportion of inhibitory failures characterized by unsmooth trajectories observed in the cued GNG task may reflect the competition between conflicting motor tendencies (go vs. no-go) during movement preparation. Another possibility might be that the higher proportion of non-one-shot movements observed in GNG inhibitory failures could reflect movement correction attempts. Proactive control promotes the maintenance of task goals; this activity serves as a source of top-down control which optimizes bias attention, perception and action systems in a goal-driven manner [2]. On this basis, it can be hypothesized that proactive control may also prompt networks dedicated to movement monitoring. Irregular and asymmetrical multi-peaked velocity profiles often characterize limb movements under constraints of time and spatial accuracy; these trajectory irregularities have been interpreted as online corrections of motor commands [49,52]. Our finding of unsmooth profiles during erroneous movements may as well reflect corrections over movement trajectories which did not corresponds to task goals. In these terms, the high proportion of non-one-shot movements in the GNG suggests that proactivity may contribute to foster mechanisms of action control over inhibitory failures.

Although previous studies [7,8,9,10,11] support the hypothesis that GNG mainly involves proactive control whereas the reactive component is mainly engaged in the SST, to further sustain this hypothesis we have to exclude the influence of other variables. In particular, discussing our findings, we should exclude that the high one-shot proportion in SST task does not depend on the difference in difficulty of inhibiting the response between the two tasks (greater in the SST than GGN task), or on the different timing in which the inhibition process occurs (delayed in the SST task), as assumed by the Independent Race Model. These aspects should be further explored in future investigations.

Furthermore, RT analysis revealed that non-one-shot movements were always slower than one-shot movements, independent of the task. This finding may be interpreted as a consequence of proactivity. Indeed, a cost in terms of RT has been defined as a characteristic feature of proactive action; it reflects the time required to release proactive inhibitory control of responses [53].

Admittedly, unsmooth trajectories and longer RTs can be interpreted as the correlates of the proactive intervention over inhibitory failures. This result is consistent with Verbruggen and colleagues [54] who indicated that proactive adjustments may act on different stages of the response inhibition process—signal detection, response selection and suppression of the motor output—to prevent wrongfully implemented motor plans. In this perspective our results provide additional information on the intervention of proactive mechanisms when the motor output has been wrongfully implemented.

fMRI studies have explored the neural correlates of proactive and reactive inhibitory processes. In particular, it has been reported that the proactive, differently from the reactive process, besides the Anterior Cingulate Cortex and the Insulae, recruits more subcortical structures including the Thalamus, the Midbrain and Cerebellum-Crus I [19,55,56,57]. According to a proposed proactive–reactive model of Gavazzi and colleagues [58], some of the above listed subcortical brain regions characterize the excitatory component of the proactive network, whereas the right inferior and middle frontal cortices would be responsible for the inhibitory component of proactive and reactive networks, respectively.

Along this line, we can hypothesize that the corrections measured during task performance in the present study might be due to the involvement of some of the above-mentioned subcortical structures [53], supporting the view of distinct motor tendencies. In particular, the midbrain and the cerebellum might be the key structures to be investigated to better understand our results, because they are involved in correcting ongoing motor plans and in the inhibitory processes [59,60,61].

These findings provide the first evidence on the possibility of distinguishing the inhibitory processes related to GNG and SST by classifying movement profiles as one-shot or non-one-shot based on the presence of trajectory corrections during inhibitory failures. This work added further insight to the body of the literature which reports a differentiation between these tasks and the involved cognitive control mechanisms [5,62,63]. Additional investigations are needed to better characterize these movement profiles by quantitative kinematic analyses. Moreover, in the present study we considered as inhibitory failures all the erroneous mouse movements (in the No-go/Stop conditions) exceeding the pre-determined threshold value, regardless of the reaching of the sponge barrier. Further insight in the processes underlying proactive and reactive inhibitory control will be gained by evaluating movement profiles of partial errors. For this purpose it will be also necessary to optimize the error rate in the GNG task in order to learn more about proactive inhibitory control by analyzing movement profiles.

The proactive component of response inhibition has been often evaluated by modified versions of the SST, where cues are inserted to inform subjects about the probability of an upcoming stop-signal, as in the conditional SST [64], or in the stop-signal anticipation task [65]. However, to distinguish reactive and proactive processes within the same task, as well as to isolate them from other cognitive process that likely modulate response inhibition (e.g., attention and working memory load), has proven difficult. Therefore, in the present study we opted to employ two separate tasks in order to maximize the link between our features of interest (i.e., velocity profile) and the respective inhibitory control component mainly involved in each task.

Other approaches have proven useful in revealing the proactive aspects of response inhibition. Namely, the reaching arm version of the stop-signal paradigm [66,67] allowed the investigation of the motor strategy optimization with respect to context demands (‘context effect’) [68]. The mouse-tracking approach, allowing the characterization of motor responses continuously over time, may provide information complementary to that obtained in the ‘context effect’ framework.

To conclude, it has been shown that under proactive circumstances (GNG), erroneous responses are characterized by typical unsmooth velocity profiles. We speculate that this might be because of conflicting motor tendencies due to proactive interactions between sensorimotor reactivity and inhibition state. Another possibility might be that proactive control triggers a higher motor control level which is then engaged in correction over movement profiles.

These results have been achieved thanks to a registration method capable of revealing information that typical button response systems cannot detect. For this reason, our findings may drive future studies aimed at better exploring reactive and proactive control in healthy subjects as well as in several special populations. As such, the implementation of this procedure which characterizes the movement profile of responses could allow the more thorough investigation of neurocognitive modifications in health and disease. Motor response inhibition mechanisms regulating sport expertise in athletes performing in different disciplines represents a relevant field of application [69]. Moreover, since a relationship between neurocognitive deficits and sport injuries has been established, the use of sensitive approaches to assess the neurocognitive status of an athlete may open new avenues to identify athletes at risk of sport injuries [70,71]. Finally, altered inhibitory control characterizes several psychopathological conditions [4]. Notably, within this category, pathological gambling has been shown to be associated with impulsivity-related personality traits and inhibitory dyscontrol [72]. However, a recent work by Sharif-Razi and coworkers did not reveal any differences in proactive and reactive inhibitory mechanisms between gamblers and healthy controls [73]. As suggested by the authors, the development of optimal tasks or new methods to assess reactive and proactive control mechanisms may impact positively in capturing the role of inhibitory processes in some psychopathological conditions. The exploration of velocity profiles extrapolated from mouse trajectories during cued GNG and SST tasks may represent one of these methods.

## Figures and Tables

**Table 1:** Behavioral performance (mean and standard deviation) for both cued GNG and SST.

	Cued GNG			SST
	Total	High GO-Stimulus	Low GO-Stimulus	
Go conditions				
Correct responses (%)	98.8 ± 5.0	98.6 ± 7.1	99.1 ± 2.4	99.4 ± 3.7
Reaction times (ms)	404.3 ± 42.2	400.2 ± 41.8	414.1 ± 45.2	411.8 ± 49.7
No-go/Stop conditions				
Inhibitory failures (%)	8.1 ± 7.8	10.0 ± 10.7	7.2 ± 7.6	74.1 ± 11.9
Reaction times (ms)	–	–	–	394.7± 41.6
SSD (ms)	–	–	–	162.0 ± 72.3

**Table 2:** Percentage of one-shot movements for both cued GNG and SST. Mean and standard deviation for the percentage of one-shot movements in Go and No-Go/stop (inhibition failures) conditions for both the cued GNG and SST.

	One-Shot Movements (%)
Go conditions	
GNG (high GO-stimulus)	97.6 ± 3.5
GNG (low GO-stimulus)	94.3 ± 15.7
SST	97.9 ± 2.1
No-go/Stop conditions (inhibition failures)	
GNG (high GO-stimulus)	21.2 ± 34.2
GNG (low GO-stimulus)	30.1 ± 33.5
SST	81.3 ± 9.1

**Table 3:** Reaction times. Mean and standard deviation for reaction times (ms) recorded for one-shot and non-one-shot movements in Go and No-Go/stop (inhibition failures) conditions for both the cued GNG and SST.

	One-Shot Movement	Non-One-Shot Movement
Go conditions		
GNG (high GO-stimulus)	401.8 ± 69.9	387.7 ± 71.0
GNG (low GO-stimulus)	417.7 ± 74.6	392.4 ± 60.8
SST	411.9 ± 95.7	412.6 ± 106.2
No-go/Stop conditions (inhibition failures)		
GNG (high GO-stimulus)	403.4 ± 67.6	435.4 ± 150.2
GNG (low GO-stimulus)	433.8 ± 120.1	498.8 ± 182.5
SST	383.4 ± 65.1	430.6 ± 106.6

## Supplementary Materials

The following are available online at https://www.mdpi.com/2076-3425/10/7/464/s1. Supplementary Materials Video S1: cued Go/No-Go, Video S2: Stop Signal Task
